# Declining amenable mortality: a reflection of health care systems?

**DOI:** 10.1186/s12913-017-2708-z

**Published:** 2017-11-15

**Authors:** Maria Michela Gianino, Jacopo Lenzi, Maria Pia Fantini, Walter Ricciardi, Gianfranco Damiani

**Affiliations:** 1 Department of Public Health Sciences and Pediatrics, Università di Torino, Via Santena 5 bis -, 10126 Turin, Italy; 2 Department of Biomedical and Neuromotor Sciences, Alma Mater Studiorum - Università di Bologna, Via Ugo Foscolo 7 -, 40123 Bologna, Italy; 3 Department of Public Health, Università Cattolica del Sacro Cuore, Largo Agostino Gemelli 8 – 00168, Rome, Italy

**Keywords:** Amenable mortality, Healthcare systems, European countries

## Abstract

**Background:**

Some studies have analyzed the association of health care systems variables, such as health service resources or expenditures, with amenable mortality, but the association of types of health care systems with the decline of amenable mortality has yet to be studied. The present study examines whether specific health care system types are associated with different time trend declines in amenable mortality from 2000 to 2014 in 22 European OECD countries.

**Methods:**

A time trend analysis was performed. Using Nolte and McKee’s list, age-standardized amenable mortality rates (SDRs) were calculated as the annual number of deaths over the population aged 0–74 years per 100,000 inhabitants. We classified health care systems according to a deductively generated classification by Böhm. This classification identifies three dimensions that are not entirely independent of each other but follow a clear order: the regulation dimension is first, followed by the financing dimension and finally service provision. We performed a hierarchical semi-log polynomial regression analysis on the annual SDRs to determine whether specific health care systems were associated with different SDR trajectories over time.

**Results:**

The results showed a clear decline in SDRs in all 22 health care systems between 2000 and 2014 although at different annual changes (slopes). Regression analysis showed that there was a significant difference among the slopes according to provision dimension. Health care systems with a private provision exhibited a slowdown in the decline of amenable mortality over time. It therefore seems that ownership is the most relevant dimension in determining a different pattern of decline in mortality.

**Conclusions:**

All countries experienced decreases in amenable mortality between 2000 and 2014; this decline seems to be partially a reflection of health care systems, especially when affected by the provision dimension. If the private ownership is maintained or promoted by health systems, these findings might be considered when thinking about regulation policies to control factors that might influence health care performance.

**Electronic supplementary material:**

The online version of this article (10.1186/s12913-017-2708-z) contains supplementary material, which is available to authorized users.

## Background

Amenable mortality is defined as premature death from a set of conditions that should not occur in the presence of timely and effective health care [1].

Adequate health care may prevent mortality due to a variety of causes by means of preventive or therapeutic measures [2].

This concept was originally developed by Rutstein et al., who created a list of conditions that were considered either treatable or preventable based on current medical knowledge and technology [3]. Subsequently, the concept of amenable mortality was explored widely, especially in Europe [4–7] and has been adopted as an indicator of the performance of health care systems by organizations such as the England Department of Health [8] and the Organisation for Economic Cooperation and Development (OECD) [8].

Levels and trends of amenable mortality have been widely documented [2, 9–13]. Most researchers have shown that levels of amenable mortality have substantially decreased over the past years. Nolte and McKee [2] conducted a comprehensive study in 19 OECD countries between 1997/1998 and 2002/2003 and found a reduction in amenable mortality in all countries. The average reduction rate was 14% in females and 17% in males. More recently, Gay et al. [9] measured the average annual change in amenable mortality in 31 OECD countries between 1997 and 2007 and concluded that amenable mortality declined in all OECD countries; the average annual decline was 3.7%.

Some studies have analyzed the associations of health care systems variables, such as health service resources [7] or expenditures, with amenable mortality [14], but the association of types of health care systems with the decline of amenable mortality has yet to be studied.

The present study builds on the aforementioned findings and examines whether specific health care system types are associated with different time trend declines in amenable mortality from 2000 to 2014. The study includes 22 OECD European countries that are associated with different types of health care systems.

## Methods

A time trend analysis was performed using secondary data from 22 European OECD countries during the period 2000 to 2014.

The mortality and population data for this study came primarily from the *World Health Organization (WHO) Mortality Database* [15], in which causes of death are coded according to the ICD-9 or ICD-10. If reference populations were not available in the WHO Mortality Database, the data were extracted from the *2012 Revision* of the *World Population Prospects* (WPP) [16] (see Additional file 1 for the list of countries included in the study).

Nolte and McKee [2, 17, 18] and Tobias and Yeh [19] prepared two different lists of causes of death that are amenable to health care. These two lists were used by the OECD to generate estimates of amenable mortality for 31 countries [9]. After reviewing the two sets of estimates of amenable mortality for the OECD countries provided by Nolte and McKee’s and Tobias and Yeh’s lists, we decided to choose Nolte and McKee’s because it provides, on average, more conservative figures (see Additional file 2, which includes Nolte and McKee’s list of causes of death amenable to health care).

Many health care system classifications exist. We adopted the typology that was presented by Rothgang and Wendt [20, 21] and modified by Böhm [22] because it attempts a deductive construction of health care system types and allows for a more precise classification of health care systems. The health care system is defined by three dimensions that are not entirely independent of each other but follow a clear order: the regulation dimension is first, followed by the financing dimension and finally service provision. In every dimension, three actors can play a role: state, societal or private actors (see Additional file 3 for a summary of Böhm’s classification) [22].

For each country, age-standardized amenable mortality rates (amenable SDRs) were calculated as the annual number of deaths in the population aged 0–74 years per 100,000 inhabitants, with direct standardization to the 2010 OECD population. First, the data were summarized by presenting the average annual amenable SDRs for the years 2000/2001 and 2013/2014 and by computing the percentage change in amenable SDRs between these time periods. Second, we performed a hierarchical semi-log polynomial regression model analysis on the annual amenable SDRs, with random intercepts and slopes to take into account individual heterogeneity across countries. In this model, we applied a log transformation of amenable SDR to ease interpretability of results (the regression slope is equal to the annual percentage change in amenable SDR) and to improve the model fit, since descriptive analysis had revealed a convex, exponential trend over time. Nevertheless, we decided to add the squared term of year to the log-linear model because the linearity assumption, checked through a joint Wald test on dummies for all years, appeared to be violated (*F*-test = 3.21, *P* = 0.003). The model included also the three health care system dimensions and their interaction with year and year-squared to determine whether specific health care systems were associated with different amenable SDR trajectories over time. Because of the limited number of countries included in the study (*n* = 22), standard errors for both fixed- and random-effects parameters were estimated using cluster bootstrapping with 1000 replicates.

For all analyses, the significance level was set at *P* < 0.05. All data were analyzed using the Stata software package, version 13 (StataCorp. 2013. *Stata Statistical Software: Release 13*. College Station, TX, USA: StataCorp LP).

## Results

**Fig. 1:**
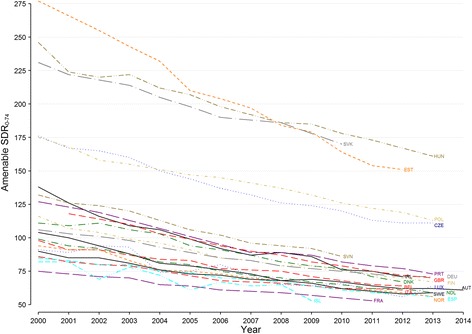
Amenable age-standardized death rates from all causes (per 100,000 people) for 0–74 year olds in 22 OECD European countries (2000 to 2014). *Note:* Missing data within the time-series of Italy (2004, 2005) and Portugal (2004–2006) were interpolated by connecting the lines between the non-missing data points. *Abbreviations:* SDR, age-standardized death rate; AUT, Austria; BEL, Belgium; CZE, Czech Republic; DEU, Germany; DNK, Denmark; EST, Estonia; FIN, Finland; FRA, France; HUN, Hungary; IRL, Ireland; ISL, Iceland; ITA, Italy; LUX, Luxembourg; NDL, Netherlands; NOR, Norway; POL, Poland; PRT, Portugal; SVK, Slovakia; SVN, Slovenia; ESP, Spain; SWE, Sweden; GBR, United Kingdom

Figure 1 displays the amenable SDRs from all causes per 100,000 persons in all 22 countries examined. All countries experienced decreases in amenable mortality between 2000 and 2014, with some relevant differences. Eastern European countries (Czech Republic, Estonia, Hungary, Poland, Slovakia) exhibited disharmonious declines and differed with a wider range of initial and final mortality rates. The other European countries exhibited small within-region differences in the reduction of amenable SDRs.

Table 1 shows amenable SDRs from all causes for the years 2000/2001 and 2013/2014. Between 2000/2001 and 2013/2014, mortality rates decreased by between approximately 30 and 45% in all countries. Table 1 also shows the distribution of the three health care system dimensions: 8 countries had statal regulation, financing and provision (Denmark, Finland, Iceland, Norway, Portugal, Spain, Sweden, United Kingdom), while other 8 countries had statal regulation, societal financing and private provision (Belgium, Czech Republic, Estonia, France, Hungary, Netherlands, Poland, Slovakia); of the remaining 6 countries, three had societal regulation, societal financing and private provision (Austria, Germany, Luxembourg), two had statal regulation, statal financing and private provision (Ireland, Italy) and one, Slovenia, had societal regulation, societal financing and statal provision.

**Table 1:** Amenable age-standardized death rates (per 100,000 people) for ages 0–74 from all causes in 22 OECD European countries, 2000/2001 and 2013/2014 (or last available years)

Country	Regulation		Financing		Provision		SDR_0–74_	SDR_0–74_	% change
	ST	SO	ST	SO	ST	PR	2000/2001^a^	2013/2014^b,c,d,e,f^	2013/2014–2000/2001
	(*n* = 18)	(*n* = 4)	(*n* = 10)	(*n* = 12)	(*n* = 9)	(*n* = 13)			
Austria		✓		✓		✓	102	62	−39.2
Belgium	✓			✓		✓	95	65	−31.6
Czech Republic	✓			✓		✓	172	111	−35.5
Denmark	✓		✓		✓		110	69	−37.3
Estonia	✓			✓		✓	272	153	−43.8
Finland	✓		✓		✓		112	69	−38.4
France	✓			✓		✓	74	54	−27.0
Germany		✓		✓		✓	105	71	−32.4
Hungary	✓			✓		✓	235	164	−30.2
Iceland	✓		✓		✓		82	59	−28.0
Ireland	✓		✓			✓	132	73	−44.7
Italy	✓		✓			✓	84	62	−26.2
Luxembourg		✓		✓		✓	90	60	−33.3
Netherlands	✓			✓		✓	97	61	−37.1
Norway	✓		✓		✓		93	58	−37.6
Poland	✓			✓		✓	172	116	−32.6
Portugal	✓		✓		✓		125	75	−40.0
Slovakia	✓			✓		✓	227	174	−23.3
Slovenia		✓		✓	✓		129	89	−31.0
Spain	✓		✓		✓		84	57	−32.1
Sweden	✓		✓		✓		88	59	−33.0
United Kingdom	✓		✓		✓		116	71	−38.8

*Notes:* Ticks indicate the type of regulation, financing and provision for each country

*Abbreviations: SDR*, age-standardized death rate; *ST*, statal; *SO*, societal; *PR*, private

^a^Amenable SDR 2001/2002 (data not available for 2000) for United Kingdom

^b^Amenable SDR 2012/2013 (data not available for 2014) for Czech Republic, Finland, Germany, Hungary, Luxembourg, Netherlands, Norway, Poland, Portugal, Spain, Sweden, United Kingdom

^c^Amenable SDR 2011/2012 (data not available for 2013/2014) for Belgium, Denmark, Estonia, Ireland, Italy

^d^Amenable SDR 2010/2011 (data not available for 2012/2014) for France

^e^Amenable SDR 2009/2010 (data not available for 2011/2014) for Slovakia and Slovenia

^f^Amenable SDR 2008/2009 (data not available for 2010/2014) for Iceland

Results of regression analysis are presented in Table 2. The downward trend of amenable SDRs, equal to 4% per year, was significant (*b* = −0.040; 95% CI = −0.043, −0.037; *P* < 0.001) and differed among the three dimensions under study: in countries with societal regulation, statal financing and private service provision the curvilinear (U-shaped) trend was more pronounced than in countries with statal regulation, societal financing and statal provision, respectively (regulation: *b* = 0.001; 95% CI = < 0.001, 0.002; *P* = 0.010; financing: *b* = −0.001; 95% CI = −0.002, −0.001; *P* < 0.001; provision: *b* = 0.001; 95% CI = 0.001, 0.002; *P* < 0.001). Of note, the standard deviation of random intercepts was significant [SD(Country) = 0.293; 95% CI = 0.189, 0.454; *P* < 0.001], indicating that the variables included in the model explain only part of the differences in SDRs among countries.

**Table 2:** Results of hierarchical semi-log polynomial regression models on amenable age-standardized death rates from all causes (per 100,000 persons) in 22 OECD European countries (2000 to 2014)

Variable	Regression	Bootstrap	*P*	Normal-based
	coefficient (*b*)	standard error		95% CI
Year	−0.040	0.002	<0.001	−0.043, −0.037
Year^2^	<0.001	<0.001	0.538	> − 0.001, 0.001
Societal regulation (ref. Statal)	−0.420	0.179	0.019	−0.732, −0.040
Societal financing (ref. Statal)	0.505	0.206	0.014	0.092, 0.865
Private provision (ref. Statal)	−0.085	0.123	0.493	−0.306, 0.186
Societal regulation × Year	−0.003	0.004	0.467	−0.011, 0.004
Societal financing × Year	0.003	0.007	0.708	−0.010, 0.017
Private provision × Year	0.002	0.007	0.807	−0.012, 0.012
Societal regulation × Year^2^	0.001	<0.001	0.010	<0.001, 0.002
Societal financing × Year^2^	−0.001	<0.001	<0.001	−0.002, −0.001
Private provision × Year^2^	0.001	<0.001	<0.001	0.001, 0.002
Constant	4.393	0.053	<0.001	4.295, 4.506
Random-effects parameters				
SD(Country-year)	0.026	0.005	<0.001	0.018, 0.039
SD(Country)	0.293	0.065	<0.001	0.189, 0.454
SD(Year)	0.006	0.002	<0.001	0.003, 0.010
SD(Year^2^)	<0.001	0.002	0.505	n/a

*Abbreviations: SDR*, age-standardized death rate; *95% CI*, 95% confidence interval; *SD*, standard deviation; n/a, not available

*Notes:* The quadratic term, year^2^, indicates the presence of a curvilinear (or nonlinear, U-shaped) trend over time. When year^2^ is positive and the linear term or slope, year, is negative, the trend is decreasing and slightly convex. The interaction terms (marked with the sign “×”) indicate how much year and year^2^ are different for different health care system types. So, year and year^2^ represent the linear and quadratic slope when regulation, financing and provision are issued by public actors, i.e., the reference category for each dimension. To obtain the linear decline in amenable SDRs for, say, countries with a societal regulation system, it is necessary to add the slope and the corresponding interaction term (−0.040–0.003 = −0.043). The exponential of the last term, constant, represents the average amenable SDR value for countries with public regulation, financing and provision (*e* ^4.393^ = 88.88)

**Fig. 2:**
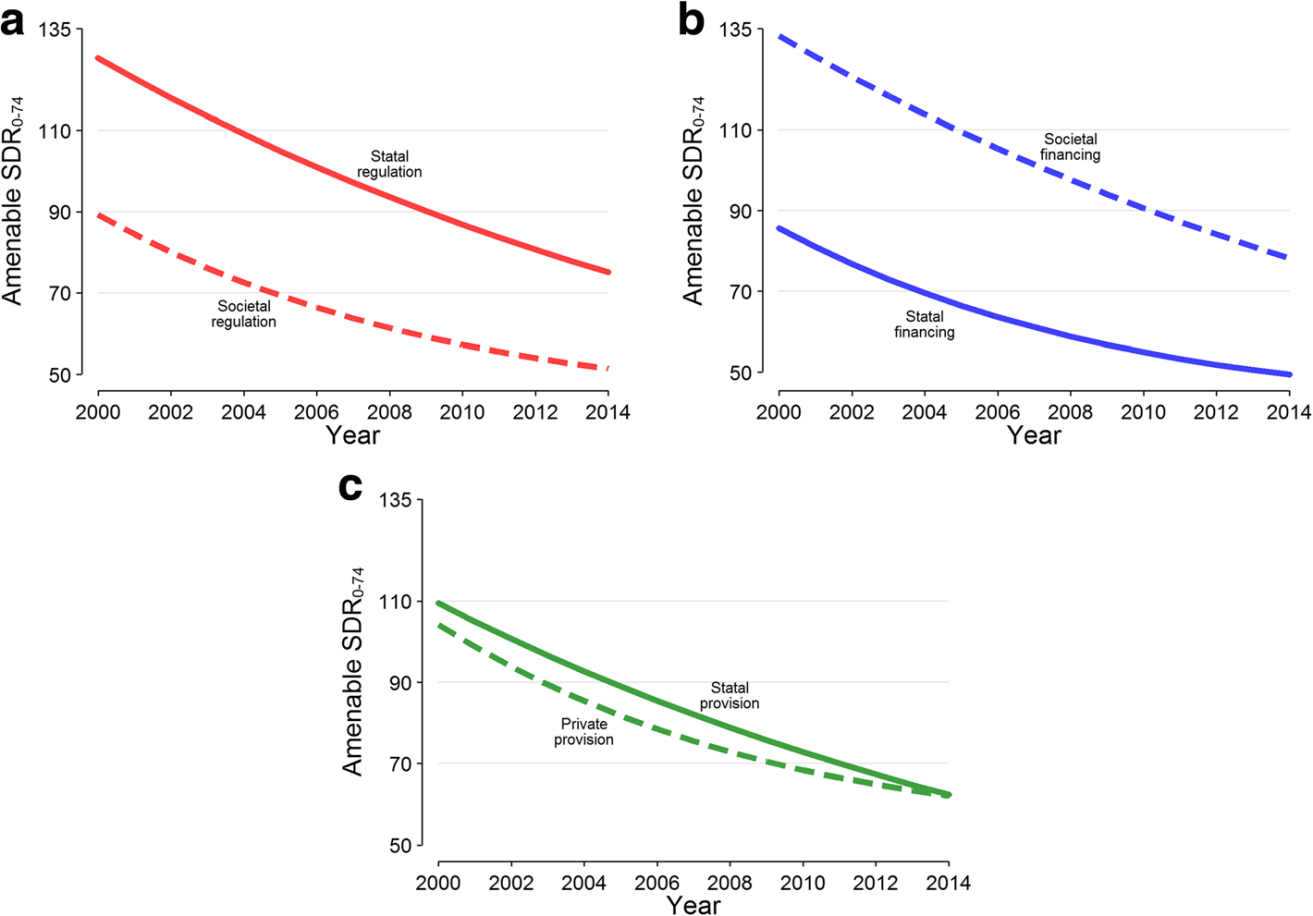
Estimated annual change in amenable age-standardized death rates (per 100,000 people) from all causes, stratified by type of regulation (**a**), financing (**b**) and provision (**c**) (2000 to 2014). *Abbreviations:* SDR, age-standardized death rate

Results of this regression analysis are also illustrated in Fig. 2, where mortality rates estimates are stratified by dimension and plotted for each year. Countries with societal regulation and statal financing had a pronounced slowdown in the reduction of amenable SDRs, but came also from low mortality levels and had presumably less room for improvement during the study period (Fig. 2a and b). The same cannot be said for countries with private provision, whose amenable SDRs at the beginning of the observation period did not significantly differ from those of countries with statal provision.

## Discussion

This study aimed to examine whether specific health care system types are associated with different patterns of decline in amenable mortality from 2000 to 2014 in 22 OECD European countries.

Due to the absence of any accepted or authoritative taxonomy of health care systems, we had to make a choice. The most used classification by researchers is OECD classification. This classification is based on the extent of coverage, mode of financing and delivery of health care, and distinguishes among three types of health systems: National Health Service (NHS), social health insurance (SHI) and the private health insurance (PHI). The NHS model combines universal coverage with funding from general tax revenue. Delivery is characterized by public ownership. The SHI model features universal coverage, and it is funded mainly by contributions and public or private delivery. Finally, in the PHI model, coverage is based on private insurance only, which is also the major funding source, and private ownership of the health infrastructure.

As highlighted by some authors, the standard trichotomous classification of health systems into national health services, social insurance systems and private insurance systems shows inherent weaknesses [23]. There are four reasons for these weaknesses. First, this classification into three ideal types is inadequate to properly describe all health systems because there is a tendency of convergence from distinct types toward mixed types of health care systems [20, 24]. Second, and consequently, the extent of coverage is not a variable that connotes health care systems because most European countries have achieved universal (or near-universal) coverage of health care [25, 26]. Third, this sort of classification is binary: a case is deemed either to belong to a specified category, or it is not. However, a set of binary decisions is reduced to one as a result of the priority given to the financing mode. Lastly, an effect of the specific attention paid to financing is the relative paucity of attention given to the matter of regulation [23].

In addition to these reasons, other reasons justify why we chose the classification proposed by Böhm [22]. The first reason for choosing Böhm’s classification was the presence of multiple dimensions (Regulation, Financing and Provision). Second, the classification provided a deductive approach to build the classification that overtakes the inductive approach, which is more or less closely related to a sample of real cases of health care systems [22]. Third, the health care sectors for this classification were weighted in the provision dimension (typically inpatient care, outpatient care and pharmaceuticals), which better combines with the concept of amenable mortality. In the present study, we found a clear decline in amenable SDRs in all 22 European health care systems between 2000 and 2014, although this decline occurred at different annual changes (or slopes). This result is consistent with previous studies and confirms that previously documented trends are continuing up to 2014 [2, 27–29].

The hierarchical regression model showed that there was a significant difference between slopes according to the provision dimension, and this result could not be ascribable to different levels of mortality at the beginning of the study period as opposed to the other two dimensions. Health care systems with a private provision exhibited a slowdown in the decline of amenable mortality over time. It therefore seems that, among the three dimensions here considered, ownership is the relevant in determining a different pattern of mortality reduction.

This finding suggests that private for-profit providers are not able to achieve additional gains in the determinants of health care improvements. These determinants include the innovation and speed of their implementation as well as the quality of care and health care coverage [13]. This interpretation gains some support from conclusions of previous studies, which note that there is little evidence to support that private for-profit providers will increasingly adopt levels of innovation and technologies. Indeed, private for-profit providers may have fewer resources to spend on care because of taxes and their over-emphasized cost control, which aims to achieve the highest possible return on investment. Consequently, this can result in less qualified staff and/or less investment on equipment or technology and can negatively impact health care related performance [30, 31]. Moreover, this interpretation is confirmed by the results of systematic reviews. Some authors [31, 32] found that the private for-profit ownership of hospitals, in comparison with private non-profit and public ownership of hospitals, results in a higher risk of death for patients and worst results on health outcomes.

Our study has strengths and weaknesses. Some studies have tried to find some relationship between health care resources or expenditure and amenable mortality. We examined whether different time trends in amenable mortality were associated with health care system type and found a relationship with the provision dimension. These findings must be interpreted very carefully; they do not suggest the superiority for one model of ownership over other. Instead, if the private for-profit ownership is maintained or promoted by health systems, these results might be considered when thinking about regulation policies that could diminish or control the negative consequences.

Of note, we investigated whether the mortality decline, and not the average amenable SDR values calculated over the study period, was affected by the three dimensions. This is why a significant difference among the average amenable SDRs by dimension can be biased by confounding factors such as national mortality levels.

The main weakness of this study is that this analysis has been conducted at the health systems dimensions level and has not disaggregated Financing, Regulations and Provision by country. Thus, findings showed that countries with private provision have seen a slowdown in the decline in mortality rates in comparison with countries with statal provision, but findings can conceal potential variation in the rate of decline among the 22 European countries. Consequently, analysis at the national level must be conducted to assist policymakers to make better-informed decisions about arrangements within providers in health care systems.

Second, the type of classification accounts for the regulation dimension which has remained a largely under-explored aspect of health care system [33].

However, for classifying the regulation dimension the basic question is “who is in charge of regulating and controlling these relationships?” but on its own it cannot explain the modes of interaction for each object in a given country. Consequently, specific aspects such as evaluation of compliance to quality standards into regulation of access of (potential) providers to health care markets (see Additional file 3) are not accounted for. These aspects may have explanatory power on level and trend of amenable mortality.

## Conclusion

This study, which is based on a time trend analysis, is an explorative research on association between specific health care systems with different SDR trajectories over time.

The main findings of our study show that the declining trend of amenable mortality rates is continuing in 2013/2014 in 22 European countries and that the decline in amenable mortality seems to be partially a reflection of health care systems, especially when affected by the provision dimension: countries with private provision saw a slowdown in the decline of mortality rates.

These findings are a source of inspiration to stimulate further researches with lower aggregated data that could overcome the limitations of this study and suggest that monitoring and evaluating the ownership of health care providers should always be considered in order to better understand the effect of one or other type of ownership. If the private ownership is maintained or promoted by health systems, these findings might be considered when thinking about regulation policies to control factors that might influence health care performance.

## Additional files

Additional file 1: List of 22 European OECD countries. (PDF 18 kb)

Additional file 2: Nolte and McKee’s list of causes of death considered amenable to health care. (PDF 24 kb)

Additional file 3: Böhm’s health care system classification: a summary. (PDF 14 kb)

Additional file 4: Primary data analyzed for this study. (XLS 46 kb)

## Abbreviations

95% CI: 95% confidence interval
AUT: Austria
BEL: Belgium
CZE: Czech Republic
DEU: Germany
DNK: Denmark
ESP: Spain
EST: Estonia
FIN: Finland
FRA: France
GBR: United Kingdom
HUN: Hungary
IRL: Ireland
ISL: Iceland
ITA: Italy
LUX: Luxembourg
NDL: Netherlands
NHS: National Health Service
NOR: Norway
OECD: Organisation for Economic Cooperation and Development
PHI: Private health insurance
POL: Poland
PR: Private
PRT: Portugal
SD: Standard deviation
SDR: Age-standardized mortality rate
SHI: Social health insurance
SO: Societal
ST: Statal
SVK: Slovakia
SVN: Slovenia
SWE: Sweden
WHO: World Health Organization
WPP: World Population Prospects
