# Supplementary material for: Declining amenable mortality: a reflection of health care systems?
Source: BMC Health Serv Res. 2017 Nov 15;17:735. doi: 10.1186/s12913-017-2708-z (PMC5688697; doi:10.1186/s12913-017-2708-z)
Supplement: Supplementary file 1 — List of 22 European OECD countries. (PDF 18 kb) [file 12913_2017_2708_MOESM1_ESM.pdf]

List of 22 European OECD countries

Country	Years	Cause of death classification	Population data source
Austria (AUT)	2000-2014	ICD-9 (2000-2001),<br>ICD-10 (2002-2014)	WHO
Belgium (BEL)	2000-2012	ICD-10	WHO
Czech Republic (CZE)	2000-2013	ICD-10	WHO
Denmark (DNK)	2000-2012	ICD-10	WHO
Estonia (EST)	2000-2012	ICD-10	WHO
Finland (FIN)	2000-2013	ICD-10	WHO
France (FRA)	2000-2011	ICD-10	WHO
Germany (DEU)	2000-2013	ICD-10	WHO
Hungary (HUN)	2000-2013	ICD-10	WHO
Iceland (ISL)	2000-2009	ICD-10	WHO
Ireland (IRL)	2000-2012	ICD-9 (2000-2006),<br>ICD-10 (2007-2012)	WHO (2000-2009, 2011-2012),<br>UN (2010)
Italy (ITA)	2000-2003,<br>2006-2012	ICD-9 (2000-2002),<br>ICD-10 (2003-2012)	WHO
Luxembourg (LUX)	2000-2013	ICD-10	WHO
Netherlands (NDL)	2000-2013	ICD-10	WHO
Norway (NOR)	2000-2013	ICD-10	WHO
Poland (POL)	2000-2013	ICD-10	WHO
Portugal (PRT)	2000-2003,<br>2007-2013	ICD-9 (2000-2001),<br>ICD-10 (2002-2013)	WHO
Slovakia (SVK)	2000-2010	ICD-10	WHO
Slovenia (SVN)	2000-2010	ICD-10	WHO
Spain (ESP)	2000-2013	ICD-10	WHO
Sweden (SWE)	2000-2013	ICD-10	WHO
United Kingdom (GBR)	2001-2013	ICD-10	WHO

Abbreviations: WHO, World Health Organization; UN, United Nations.
