# Supplementary material for: Declining amenable mortality: a reflection of health care systems?
Source: BMC Health Serv Res. 2017 Nov 15;17:735. doi: 10.1186/s12913-017-2708-z (PMC5688697; doi:10.1186/s12913-017-2708-z)
Supplement: Supplementary file 2 — Nolte and McKee’s list of causes of death considered amenable to health care. (PDF 24 kb) [file 12913_2017_2708_MOESM2_ESM.pdf]

Nolte and McKee's list of causes of death considered amenable to health care

Disease categories	Age	Diseases	ICD-9-CM codes	ICD-10-CM codes
Infectious diseases	0–74	Tuberculosis	010–8, 137	A15–9, B90
	0–74	Septicemia	38	A40–1
	0–74	Pneumonia	480–6	J12–8
	0–74	Influenza	487	J10–1
	0–14	Intestinal infections (other than typhoid, diphtheria)	001–9	A00–9
	0–74	Diphtheria, Tetanus, Poliomyelitis	032, 037, 045	3A35–6, A80
	0–14	Whooping cough	33	A37
	1–14	Measles	55	B05
Cancers	0–74	Colorectal cancer	153–4	C18–21
	0–74	Malignant neoplasm of skin	173	C44
	0–74	Breast cancer	174	C50
	0–44	Cervical cancer and uterine cancer	179, 180, 182	C53–5
	0–74	Neoplasm of the testis	186	C62
	0–74	Hodgkin's disease	201	C81
	0–44	Leukemia	204–8	C91–5
Endocrine, nutritional and metabolic diseases	0–74	Thyroid disorders	240–6	E00–7
	0–49	Diabetes mellitus	250	E10–4
Diseases of the nervous system	0–74	Epilepsy	345	G40–1
Diseases of the circulatory system	0–74	Rheumatic heart diseases	393–8	I05–9
	0–74	Ischemic heart diseases: 50% of deaths	410–4	I20–5
	0–74	Cerebrovascular diseases	430–8	I60–9
	0–74	Hypertensive diseases	401 – 5	I10–3, I15
Diseases of the genitourinary system	0–74	Nephritis and nephrosis	580–9	N00–7, N17–9, N25–7
	0–74	Benign prostatic hyperplasia	600	N40
Diseases of the respiratory system	1–14	All respiratory diseases (excl. pneumonia/influenza)	460–79, 488–519	J00–9, J20–99
Diseases of the digestive system	0–74	Peptic ulcer	531–3	K25–7
	0–74	Appendicitis	540–3	K35–8
	0–74	Abdominal hernia	550–3	K40–6
	0–74	Cholelithiasis and cholecystitis	574–5.1	K80–1
Perinatal mortality	0–74	Maternal deaths	630–76	O00–99
	0–74	Perinatal deaths (excl. stillbirths)	760–79	P00–96
	0–74	Congenital cardiovascular anomalies	745–7	Q20–8
External causes	0–74	Misadventures to patients during surgical and medical care	E870–6, E878–9	Y60–9, Y83–4

Source: Nolte and McKee 2088; Gay et al. 2011.
