# Supplementary material for: Declining amenable mortality: a reflection of health care systems?
Source: BMC Health Serv Res. 2017 Nov 15;17:735. doi: 10.1186/s12913-017-2708-z (PMC5688697; doi:10.1186/s12913-017-2708-z)
Supplement: Supplementary file 3 — Böhm’s health care system classification: a summary. (PDF 14 kb) [file 12913_2017_2708_MOESM3_ESM.pdf]

# Böhm's health care system classification: a summary

| Dimensions | Taxonomy | Classification mode of healthcare systems |
|---|---|---|
| **Financing** | **Private:** contributions to private insurance and out of pocket payments<br>**Societal:** social security funds<br>**State :** general tax revenues | The highest share determines in which group the respective country is classified. |
| **Regulation** | **Private**<br>**Societal**<br>**State**<br>Accordig to actors respnsible for regulating the object in question.<br>The objects are:<br>**Relations between (potential) beneficiaries and financing agencies** :<br>*(1) System of financing*: the financing of health care by public and /or private sources<br>**Relations between financing agencies and service providers**:<br>*(2) Remuneration of service providers :* the spec fic system of provider compensation<br>*(3) Access of (potential) providers to health care markets:* access to financing agencies<br>**Relations between service providers and (potential) beneficiaries***:*<br>*(4) Access of patients to service providers:* the specific delivery of care to patients<br>*(5) Benefit package :* the content and range of services offer ed to patients. | Each object of regulation according to the actor responsible for regulating the field in question is classified.<br>As regulatory actors often differ between inpatient and outpatient sectors, both sectors separately for the regulatory fields (2)–(4) is classified.<br>After having classified each of the five categories, all of them are summarized and create one point if the whole category is defined as state, societal, or private, respectively, half a point if two different actors are relevant in one category, and one third of a point if all three actors are involved in regulation. The actor dominating the most categories determines classification. |
| **Service Provision** | **Private:** private for profit actors<br>**Societal:** private not for profit cctors<br>**State :** public actors | A trichotomous service provision index is used.<br>Steps to constructing this index:<br>The first is to allocate weights to the main health care sectors (inpatient care, outpatient care, pharmaceuticals) according to the financial resources allocated to each sector.<br>The second is to identify the shares of the dominant providers within each sector.<br>The third is to multiply the standardized share of total health spending of the selected sectors with the relative shares of private, societal, and state actors in each sector, and then sum up the received shares of private, societal, and public provision.<br>Decisive for the classification is which actor provides the relatively highest share of services. |
